# Development of Ultra-Performance Liquid Chromatography–Mass Spectrometry Method for Simultaneous Determination of Three Cationic Dyes in Environmental Samples

**DOI:** 10.3390/molecules25194564

**Published:** 2020-10-06

**Authors:** Afnan Ali Hussain Hakami, Saikh Mohammad Wabaidur, Moonis Ali Khan, Zeid Abdullah Alothman, Mohd. Rafatullah, Masoom Raza Siddiqui

**Affiliations:** 1 Chemistry Department, College of Science, King Saud University, Riyadh 11451, Saudi Arabia; 437203979@student.ksu.edu.sa (A.A.H.H.); swabaidur@ksu.edu.sa (S.M.W.); mokhan@ksu.edu.sa (M.A.K.); zaothman@ksu.edu.sa (Z.A.A.); 2 Chemistry Department, Faculty of Science, Jazan University, Jazan 45142, Saudi Arabia; 3 School of Industrial Technology, Universiti Sains Malaysia, Penang 11800, Malaysia

**Keywords:** methylene blue, rhodamine B, crystal violet, solid-phase extraction, pistachio shell biomass

## Abstract

Lower dye concentrations and the presence of several dyes along with other matrices in environmental samples restrict their determination. Herein, a highly sensitive and rapid ultra-performance tandem mass spectrometric method was developed for simultaneous determination of cationic dyes, namely methylene blue (MB), rhodamine B (RB) and crystal violet (CV), in environmental samples. To preconcentrate environmental samples, solid-phase extraction cartridges were developed by using hydrogen peroxide modified pistachio shell biomass (MPSB). The surface morphological and chemical functionalities of MPSB were well characterized. The developed method was validated considering different validation parameters. In terms of accuracy and precision, the %RSD for all three dyes at all four concentration points was found to be between 1.26 and 2.76, while the accuracy reported in terms of the recovery was found to be 98.02%-101.70%. The recovery was found to be in the range of 98.11% to 99.55%. The real sample analysis shows that MB, RB, and CV were found in the ranges of 0.39–5.56, 0.32–1.92 and 0.27–4.36 μg/mL, respectively.

## 1. Introduction

Dyes are a class of compounds encountered by humans in all aspects of life, ranging from cosmetics to clothing, food and even pharmaceuticals. Methylene blue (MB), rhodamine B (RB) and crystal violet (CV) are dyes that have a considerable number of practical applications, and they have extensively studied by researchers. Methylene blue, IUPAC name 7-(dimethylamino)phenothiazin-3-ylidene]-dimethylazanium chloride, is extensively used as a dye and is also reported to be the most primitive synthetic drug [1,2]. It is usedwhe in the textile and paper industries, office supplies, redox reactions and laboratory experiments, also finding practical application as a pH indicator. Overexposure to methylene blue has adverse effects on human health. Cyanosis, nausea, methemoglobinemia and the formation of Heinz bodies are some effects reported to be caused by MB exposure [3]. 

Rhodamine B, IUPAC name [9-(2-carboxyphenyl)-6-diethylamino-3-xanthenylidene]-diethylammoniumchloride is another important dye that has numerous applications (e.g., colorant in textile, staining, biomarker in rabies vaccine for raccoons, food industry applications, and biotechnological applications [4,5,6]). Skin irritation and respiratory tract and eye ailments are associated with RB; it is also regarded as a toxic and potentially carcinogenic substance [6,7] 

Crystal violet (CV), IUPAC name N-[4-[bis[4-(dimethyl-amino)-phenyl]-methylene]−2,5-cyclohexadien-1-ylidine]-N-methyl-methanaminiumchloride, also known as gentian violet and hexamethyl pararosaniline chloride, is a multipurpose dye that is used as a histological stain, has antimicrobial properties and was previously applied as antiseptic [8]. Like MB, CV has applications in a wide range of industries, including paper, food, paint, rubber, printing and cosmetics [9]. In addition to its beneficial applications, CV is reported to be toxic for aquatic as well as terrestrial organisms. Studies on CV suggest that it is a mitotic poisoning agent and can be considered as a biohazardous substance [10,11]. Skin and digestive tract irritation, kidney failure and respiratory ailments are commonly caused by CV exposure [12]. 

The reports available in the literature point out the usefulness of these dyes to human beings in some way or another, while also highlighting the ill effects that occur when these dyes, after their use, are discharged into water bodies through different routes including domestic sewage, black water, gray water, industrial discharge and college/university discharge. Once discharged into the water bodies, these dyes, even in small quantities, contaminate them and make them unfit for human use. These dyes also pose a threat to the aquatic life as they tend to reduce light penetration and lead to an increase of biochemical oxygen demand (BOD) and chemical oxygen demand (COD) of the water system [13,14]. Furthermore, these dyes can enter the human body through the food web and cause damage to human health. Thus, these dyes must be determined quantitatively to assess their presence in water sources. 

In the view of their importance, applications and related health issues, these dyes were quantitatively determined using different analytical instruments. Spectrophotometry, a widely used method for analyses of dyes, has been utilized in the analysis of MB [15,16,17], RB [18,19,20] and CV [21,22], although in different matrices. Electrochemical analysis has also been conducted on MB, CV and RB [23,24,25]. Chromatographic techniques, from HPLC [26,27,28,29] to LC-MS [30,31,32,33,34,35,36,37,38,39], have been intensively used to study MB, RB and CV in different matrices. Taking into consideration the ill effects of these dyes on human health, a rapid and accurate method is required to analyze them in environmental samples. For this, an ultra-performance liquid chromatography–mass spectrometry technique with solid-phase extraction was adopted. In addition to the chromatography, mass spectrometry can also be combined with capillary electrophoresis [40]. To the best of our knowledge, no UPLC-MS method has yet been reported for the simultaneous determination of these three dyes.

## 2. Materials and Methods

### 2.1. Chemicals and Reagents

Methylene blue from CDH (New Delhi) and crystal violet and rhodamine B from Sigma-Aldrich (USA) were used for the experiments. Shelled pistachio was purchased locally. Methanol, ethanol, acetonitrile, acetone, dichloromethane, formic acid and acetic acid were procured from BDH Chemical (Poole, the United Kingdom). Ammonia solution and sodium hydroxide used during various experiments were purchased from Merck (Germany). The real samples were collected from two different laundry units located in Riyadh, two different textile dyeing units and printing press waste in India, domestic supplies in Riyadh, bottled drinking water from a local store in Riyadh and irrigation supply water from Riyadh locality. 

### 2.2. Instrumentation

All the pH studies were performed on the Orion 2-star pH benchtop (Thermos Scientific, Beverly, MA, USA). The samples were weighed on a Prescica XB 220A analytical balance (Precisa Gravimetrics AG, Dietikpn, Switzerland), while the solid-phase extraction was carried out using extraction assembly (Supelco, Bellefonte, PA, USA), Extrelut extraction column (Millipore, MA, USA), coupling pieces, stopcocks and vacuum manifold. The instrumental analysis was performed using an Acquity H-Class ultra-performance liquid chromatography system (TQD) manufactured by Waters, Milford, USA. Solvent manager, quaternary pump, column heater, sample manager and Acquity UPLC BEH C18 1.7 μm, 2.1 × 50 mm column comprised the LC system, while the TQD system was equipped with electrospray ionization. The application manager QuanLynx, including MassLynx 4.1, was used for data acquisition and processing. Other important contributors to the MS system included IntelliStart for sample tuning, a rotary pump for assisting vacuum and a nitrogen generator for supplying desolvation gas and argon as collision gas from a local supplier. The characterization of the samples was done on FT-IR spectrometer, PerkinElmer 2000 (Perkin Elmer Corp, Waltham, MA, USA) XRD on a Bruker (Billerica, MA, USA) D8 advance with Cu Kα radiation in a scanning range of 5–60 2θ(with a scan rate of 12/min), while the scanning electronic micrograph (Quanta FEG 650 SEM, thermofisher, Beverly, MA, USA) was used for the surface morphological studies. 

### 2.3. Preparation of Standard Solution

The stock solution of the individual dyes was prepared in Milli-Q water (Millipore, Bedford, MA, USA), with each dye having a concentration of 5 mg L^−1^. Individual dyes were further diluted to get the desired concentrations of 32 ng mL^−1^, 64 ng mL^−1^, 125 ng mL^−1^, 250 ng mL^−1^, 500 ng mL^−1^ and 1000 ngmL^−1^; the lower linear range solutions were used to prepare the limit of detection ( LOD) and limit of quantitation (LOQ) samples. 

### 2.4. Sample Preparation 

Wastewater samples were collected from different locations. The wastewater samples included laundry waste, textile dyeing unit waste, printing press waste, domestic supplies, bottled drinking water and irrigation supply water. The samples were filtered through a syringe filter of 0.2 µm pore size prior to UPLC-MS analyses. In an attempt to evaluate the recovery of the method, standard sample was spiked into the dye-free wastewater sample, and the concentrations of the dyes in the spiked sample were calculated from the calibration plot. 

### 2.5. Development and Validation of UPLC-MS/MS and SPE Biosorbent Preparation

The development of an analytical method is just an initial step in the development process where different LC and MS parameters are optimized so that the best results can be obtained. In the current analysis, the three pollutant dyes were determined using ammonium acetate and acetonitrile (0.1% formic acid) as mobile phase, whereas the MS studies were taken up in positive ionization mode, optimizing the various MS conditions. After obtaining the best results, proper validation studies were performed considering different parameters such as linearity, precision, recovery, system suitability, limit of detection and limit of quantitation. Detailed studies on these parameters are necessary to make sure that the developed method works properly so that it can be applied for analysis of the target analyte in environmental samples.

Selection of the solid phase in solid phase extraction (SPE) is an important part of the study. Going through the literature, it was found that the pistachio shell and similar types of lignocellulosic material are used for the adsorption studies of these dyes. In the literature, pistachio shell is also reported to have a wide range of adsorptive applications, ranging from dyes to heavy metals; thus, pistachio shell was selected as the SPE material [41,42,43,44]. To prepare the solid-phase material, pistachio shells were collected and washed, followed by drying till removal of the moisture content. The dried pistachio shells were crushed manually, ground to a small size and sieved (particle size 0.2–0.4 mm). Then, 10.0 g of sieved pistachio shells was taken in a 250 mL beaker, mixed with 200 mL 10% (*w*/*w*) hydrogen peroxide and kept under continuous stirring at 100 rpm in a shaking water bath at 50 °C for 1 h. Treatment with hydrogen peroxide was carried out to decompose the organic content of the biomass and simultaneously enrich it with oxygen-containing functional groups [45,46]. After this process, the whole content was washed to make sure that even trace hydrogen peroxide was removed from the biosorbent. Then, the sorbent was dried at 80 °C for a sufficient time prior to its application. 

### 2.6. Solid-Phase Extraction Column Preparation and Experimental Conditions

For the extraction of methylene blue, rhodamine B and crystal violet from the environmental samples, an SPE procedure was adopted, where the biosorbent derived from the pistachio shell modified with hydrogen peroxide was used as a sorbent material, i.e., the solid phase in the extraction column. For preparation of the SPE column, 0.75 g of pistachio shell biosorbent was weighed and filled in between two coupling pieces in a 6 mL Extrelut extraction column. The prepared column was washed with ethanol and water to remove the impurities from the sorbent materials, followed by drying under vacuum for 10 min. Twenty-five milliliters of the wastewater was subjected to extraction by passing through the prepared solid-phase column where the flow rate was fixed at 0.75 mL/min. The column was rinsed with water to remove the traces of unabsorbed dyes and then dried under vacuum conditions. The three dyes adsorbed on the biosorbent were desorbed using 0.1% formic acid in methanol, where the volume of the eluting solvent was 30 mL. The eluted samples were then dried and preconcentrated in 2.0 mL water. 

## 3. Results and Discussion

### 3.1. Method Development and Optimization

The separation of the three dyes was archived by ultra-performance liquid chromatography–mass spectrometry (UPLC-MS). The foremost condition required while proceeding with the UPLC-MS method development is the proper selection of mobile phase so that the analytes obtain excellent peak shape and decent resolution and sensitivity, whereas retention time is yet another factor that is very important considering the time of analysis. Considering these different factors, several mobile phase combinations were tried including methanol/water, acetonitrile/water, ammonium acetate/methanol, ammonium acetate/acetonitrile, ammonium acetate/acetonitrile+formic acid, ethanol/water, formic acid/methanol and formic acid/acetonitrile. In all cases, different compositions of the two mobile phase constituents were tried. The three compounds were detected in some of the mobile phase combinations mentioned above, including ammonium acetate and acetonitrile. However, the biggest hurdle appeared in the separation of the three compounds with good peak shape and sensitivity of the three analytes. Optimizing a few other experimental conditions, such as the concentration of ammonium acetate (4 mM) and the addition of 0.1% formic acid in acetonitrile as an organic modifier, led to improved peak shape and resolution. Thus, these conditions were selected for further studies, as they were found to be most suitable among the tried mobile phases. Furthermore, to improve the sensitivity, peak shape and resolutions and to obtain the best quantitative results, different compositions of the selected mobile phase (ammonium acetate and acetonitrile (0.1% formic acid)) were tried in varied ratios ranging from 20 to 80% contribution of ammonium acetate. It was found that the mobile phase with equal contributions of ammonium acetate and acetonitrile (0.1% formic acid) resulted in the best peak, with good resolution, when the mobile phase was allowed to flow at a rate of 0.4 mL/min. Therefore, these parameters were selected for further studies.

**Figure 1:**
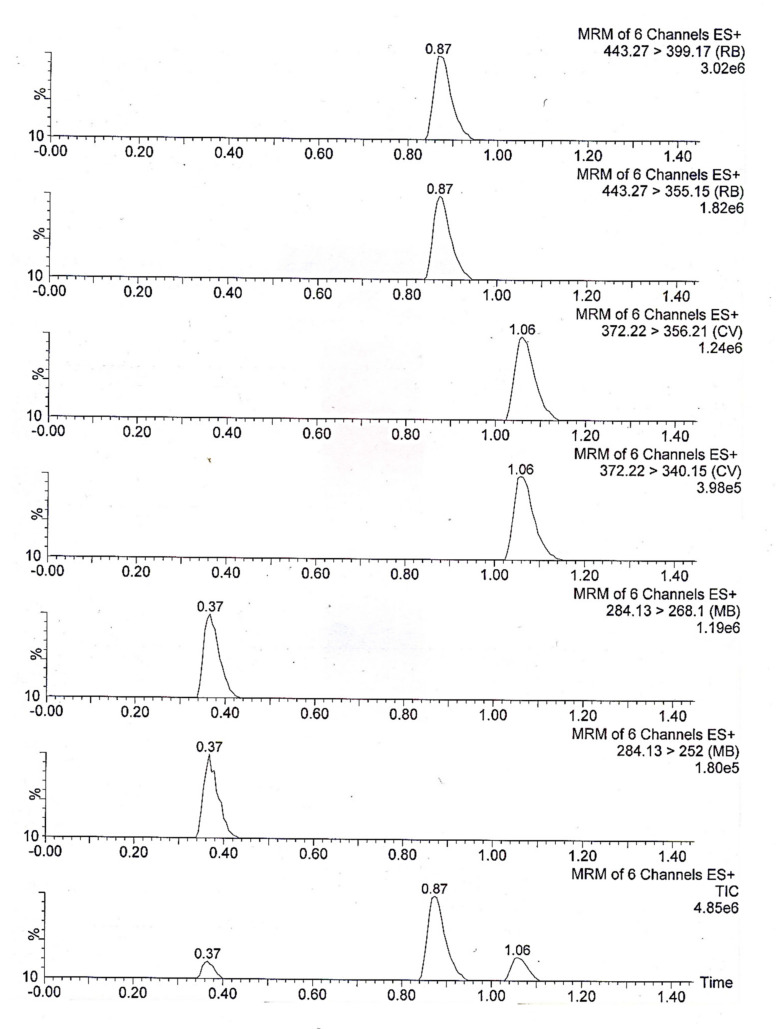
MRM chromatograms of methylene blue, rhodamine B and crystal violet using UPLC-MS/MS.

Ionization mode selection is another optimization parameter and is used for the selection of parent ions, where ESI^+^ or ESI^−^ is checked and selected based on the sensitivity and the chemical ionization characteristics of the analytes. The mass-to-charge ratio (*m/z*) of the parent ion of the individual analytes, i.e., methylene blue, rhodamine B and crystal violet, was confirmed by infusion of the combined sample of 1000 ng mL^−1^ through the syringe pump to the mass spectrometer. The obtained results suggest that methylene blue, rhodamine B and crystal violet form the [M+H]^+^ parent ion under ESI^+^ mode. The details of the MS and the analyzer parameters are given in Table 1, while the details of parent ion and the resulting daughter ions are given in Table 2. The Multiple reaction monitoring (MRM) chromatograms of the three dyes are presented in Figure 1.

### 3.2. Method Validation

Before proceeding to the application of any newly developed analytical method, the developed method should be validated to ensure that the method is correct and capable of accurately analyzing the target analytes both qualitatively and quantitatively. Here, a few parameters needed to be checked to ascertain the stability of the analyte’s peak, shape of the peak, linear range, accuracy via recovery of the samples, precision through repeatability studies and limits of detection and quantitation, where each parameter was studied individually. 

#### 3.2.1. Linearity and Range

This study was performed taking six concentration points. Six different replicates of three analytes with concentrations of 32, 64, 125, 250, 500 and 1000 ng mL^−1^ were prepared and analyzed, and a calibration graph was prepared, taking the mean of peak area at each concentration point versus the respective concentration. Within the studied points, the three dyes were found to be linear in the studied range and had the following linear regression equations: A = −14,715+1165.5×C (methylene blue); A = 20,526 + 1579.2 × C (rhodamine B) and A = 31,620 + 2565.3 × C (crystal violet). The R^2^ for the three dyes was found to be in the range of 0.9997–0.9999.

#### 3.2.2. Accuracy and Precision

Precision studies were conducted taking four concentration points for each dye; these concentration points were lower, middle, and higher points within the linear range. The concentrations considered were 32, 125, 500 and 1000 ng mL^−1^. The precision was evaluated in terms of repeatability, while the accuracy was measured in terms of the recovery. The accuracy and precision studies are summarized in Table 3, which reveals that the % RSD at all four concentration points for intraday precision for methylene blue was found in the range of 1.26–1.82, while the interday precision for the same dye was found to be in the range of 1.60–2.02. Analyzing the rhodamine B data, the % RSD for the interday assay was found to be in the range of 1.33–1.92, while that of the intraday assay was found to be in the range of 1.99–2.25. The % RSD for the crystal violet in the intraday precision assay was found to be in the range of 1.45–1.85, while that of the interday assay was found to be in the range of 1.99–2.76. 

#### 3.2.3. Limit of Detection and Limit of Quantitation

The LOD sample was prepared by diluting the lowest concentration of the calibration range, while the LOQ sample was prepared at a concentration above the LOQ level. In addition, the blank sample was also injected. In the current study, the signal-to-noise ratio was used to evaluate the LOD and LOQ. The noise around the analyte retention time was measured, and the concentration of analyte that produces a signal equal to a certain noise value was also recorded to get information about signal and noise. An S/N ratio of 3 represented the LOD, and an S/N ratio of 10 was used to get information about the LOD. In this study, the LOD and LOQ were found to be 8 ng mL^−1^ and 25 ng mL^−1^, respectively.

#### 3.2.4. Recovery Studies

Recovery studies were conducted by spiking a known amount of the analyte solution in the analyte-free environmental sample, and the analyte amounts were calculated using the calibration curve. Four concentration points, 64, 125, 250 and 1000 ngmL^−1^, were spiked into the analyte-free samples, and the amounts of the dyes recovered using the developed procedure after solid-phase extraction were calculated. The details of the recovery studies are provided in Table 4 and show recoveries of 98.11–99.23% (% RSD 1.34–2.59) for methylene blue, 98.33–99.45% (% RSD 0.66–2.15) for rhodamine B and 99.34–99.55% (%RSD 0.67–2.82) for crystal violet. 

#### 3.2.5. Selectivity of the SPE material

Selectivity of the SPE materials was tried on anionic dyes such as tartrazine and allura red. The experiments suggest that the adsorbent in combination with the eluting solvent used in the current study is not fit for their extraction. It can be suggested that the dye is selective for these cationic dyes under the current experimental condition.

### 3.3. Extraction Procedure and Characterization

#### 3.3.1. Optimization of the Solid-Phase Extraction Method

For effective extraction of methylene blue, rhodamine B and crystal violet, the extraction conditions must be optimized to get the best results. For the optimization of the extraction process, each dye was individually adsorbed and eluted using different solvents, and the common optimum extraction conditions were established. Before the start of the extraction process, 20 mL ethanol was passed through the column for removal of impurities, followed by washing with 50 mL water at the same flow rate. To check the effect of flow rate of the eluent, 25 mL of 500 ng mL^−1^ of individual dyes was passed through the solid-phase extraction column at five different flow rates (3.0, 2.0, 1.0^1^, 0.75 and 0.5 mL min^−1^) and the percentage of dyes in the eluate was calculated. The results are provided in Table 5. The results of the quantitative analysis of the eluate reveal that the sample moving through the extraction column at flow rates of 1.0 to 3.0 mL min^−1^ has a significant amount of the target analytes, proving that the adsorption of the dyes on the biosorbent bed of the column is significantly higher at lower flow rates than that at the higher flow rate. This can be attributed to the higher residence time of dyes in the biosorbent at lower low rates. Thus, 0.75 mL/ min was selected as optimum flow rate. To get the most effective recovery of the dyes, elution of the adsorbed dyes was processed, and several solvents were tried, including methanol, acetonitrile, acetone, basic methanol and acidic methanol, with formic acid. The elution studies suggest that the best recoveries of the three dyes were achieved with acidified methanol (0.1% formic acid). Therefore, for all the recovery studies, 0.75 mL flow rate and acidified methanol (0.1% formic acid) were used

#### 3.3.2. Characterization of the Biosorbent Post- and Pre-Extraction 

**Figure 2:**
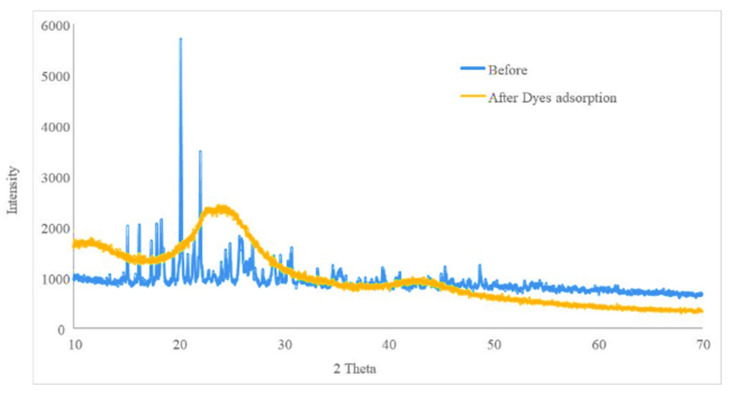
XRD pattern of the solid-phase extraction sorbent before and after the extraction.

**Figure 3:**
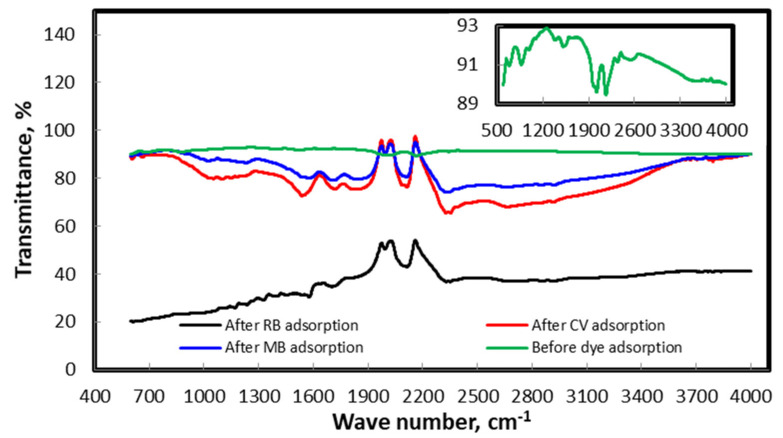
FT-IR spectra of SPE extraction sorbent material before and after extraction process. Inset: FT-IR spectrum of pristine SPE sorbent.

**Figure 4:**
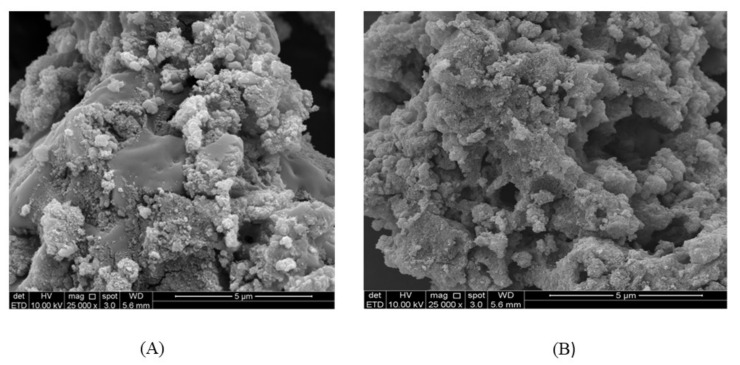
SEM images of the sorbent material: (**A**) before adsorption; (**B**) after adsorption.

Different techniques were used for the characterization of the synthesized biosorbent. FT-IR, SEM and XRD were the three techniques used in this study, and their outputs are summarized in Figure 2, Figure 3 and Figure 4. Figure 2 illustrates the XRD patterns of hydrogen peroxide modified pistachio shell (PS) biosorbent before and after adsorption of methylene blue, rhodamine B and crystal violet. The typical XRD absorption bands at 2θ of 15.1° and 22.2° are due to the presence of cellulosic structure in the materials [47]. The sharp and intense peak indicates the pure crystalline structure, which might be due to the removal of hemicellulose and lignin contents of the materials during H_2_O_2_ treatment and drying [48,49]. Figure 2 also illustrates the XRD patterns of the materials after dye adsorption. In all cases, the peaks clearly became weak and blunt after dye adsorption, indicating the amorphous nature of the dye-saturated materials. The average crystalline size of the material was calculated using the Debye-Scherrer formula and found to be 59 nm. Figure 3 illustrates the FT-IR spectra of H_2_O_2_ treated PS biosorbent before and after dye adsorption. Pristine PS biosorbent showed a band centered at 3498 cm^−1^ due to hydroxyl (–OH) group vibrations. A weak band at 1649 cm^−1^ was assigned to carbonyl (C=O) group stretching vibration. A broad and strong band ranging between 1490 and 1610 cm^−1^ was associated with C=C bending vibrations of aromatic rings present in lignin [50], while a very weak band near 1645 cm^−1^ was due to alkenyl C=C stretching. A band at 1361 cm^−1^ was assigned to bending vibrations of –OH and aliphatic deformation vibration of –CH_3_ and –CH_2_ groups present in cellulose and lignin. A band at 1058 cm^−1^ was due to –C–O stretching vibration. A band at 862 cm^−1^ was assigned to β-glucosidic linkage between the polysaccharides. After MB, RB and CV adsorption, shifting and changes in band size were observed. The strong-intensity bands at 1649 and 1058 cm^−1^ with little shifting were observed due to the electrostatic interaction between cationic dye ions and carbonyl groups present on the biomass. An intense band at 1552 cm^−1^ in dye-saturated biomass sample spectra depicts π–π stacking interactions between C=C groups present on the biomass and the aromatic rings of the dyes. Therefore, mechanistically, the bonding with the dyes on the PS biomass surface was due to electrostatic forces and π–π stacking interactions. The morphology of pistachio shell adsorbents was studied by SEM. Figure 4 shows the surface morphology of the prepared adsorbents before and after dye adsorption. Figure 4A shows an uneven surface with dust-like macroparticles. It also shows the agglomeration of many fine microparticles, which form a rough surface and cauliflower-like pore structures. The roughness of the surface that was formed might be due to the rupture of hemicelluloses and lignin [51]. After dye adsorption (4B), a clear deposition of the dyes on the surface of the adsorbent is noticed. The surface roughness and pore structures make the material suitable for sorption and increase its capability to act as a sorbent.

### 3.4. Application of the Developed Method

The developed method was applied to the simultaneous determination of the three dyes in environmental samples. Wastewater samples collected in Riyadh (from different domestic supplies, irrigation supply water, bottled drinking water and laundries) and India (from textile dyeing units and printing presses) were analyzed; the results of the determination of the studied analytes are given in Table 6. The obtained results suggest the presence of the dyes in different wastewater samples. These wastewater samples are supposed to be discharged in different water bodies. The presence of these dyes in the water bodies may lead to different health hazards. These dyes may accumulate in various human food materials such as fish and other seafood and may enter the human body through these foods, where they may create health problems. The dyes may also severely affect aquatic organisms in these water bodies. The presence of traces of these dyes may block the penetration of light, which may in turn block photosynthesis, altering physiological processes. 

## 4. Conclusions

In this study, a new UPLC-MS/MS method has been developed for the quantitative analysis of three cationic dyes, namely methylene blue, rhodamine B and crystal violet. The separation of the three dyes was achieved using 4 mM ammonium acetate and 0.1% formic acid in acetonitrile in an equal ratio. The developed method was validated considering parameters such as linearity, accuracy and precision, recovery and limits of detection and quantitation. The developed method was applied for the determination of the three dyes in environmental samples such as laundry waste, textile waste and printing press waste. Other waste samples from sources such as domestic supplies and irrigation supply water were also investigated for possible dye content. The results of the validation experiments suggest that the three dyes were linear in the concentration range of 32 to 1000 ng mL^−1^. Additionally, the accuracy and precision study suggest that % RSD at all the studied concentration points was found to be in the range of 1.26–2.76. Accuracy was evaluated in terms of percent recovery of the precision samples (both intra and interday) and was found to be between 98.02% and 101.70% at all concentration points. The method was applied to analyze the dye content in real samples. Prior to the determination process, the sample extraction process was performed using solid-phase extraction and preconcentration in 2.0 mL. In the real samples studied in the application study, methylene blue was found in the range of 0.39–5.56 µg mL^−1^, rhodamine B was found in the range of 0.32–1.92 µg mL^−1^ and crystal violet was found in the range of 0.27–4.36 µg mL^−1^. 

## Figures and Tables

**Table 1:** MS parameters for determination of methylene blue, rhodamine B and crystal violet.

S. No	MS Parameter	Value	Analyzer Parameter	Value
1	MS scan Range	205–512	LM1 Resolution	9.24
2	Capillary (kV)	3.60	HM 1 Resolution	15.00
3	Cone (V)	45.00	Ion Energy	0.26
4	Extractor (V)	3.00	MSMS Mode entrance	1.0
5	RF (V)	0.10	MSMS Collision Energy	40
6	Source Temperature	150	MSMS Exit mode	0.50
7	Desolvation temperature (°C)	350	LM 2 Resolution	10.59
8	Cone gas flow (L/H)	0	HM 2 resolution	15.00
9	Desolvation gas flow (L/h)	600	Ion energy 2	1.0
10	Collision gas flow (mL/min)	0.12	Gain	1.0

**Table 2:** Molecular formula, retention time and MRM parameters used for the determination of methylene blue, rhodamine B and crystal violet.

Analyte	Molecular Formula	Retention Time	Precursor Ion	Molecular Transition	
				Qualitative	Quantitative
Methylene Blue	C_16_H_18_ClN_3_S	0.37	284.13	252	268.1
Rhodamine B	C_28_H_31_ClN_2_O_3_	0.87	443.27	355.16	399.17
Crystal Violet	C_25_H_30_ClN_3_	1.06	372.22	340.15	356.21

**Table 3:** Accuracy and precision study for the determination of methylene blue, rhodamine B and crystal violet.

Precision	Intra-day Precision				Inter-day Precision		
	Taken ng mL^−1^	Found ng mL^−1^	RSD %	Recovery %	Found ng mL^−1^	RSD %	Recovery %
	32	31.90	1.40	99.68	32.11	1.76	100.34
Methylene Blue	125	123.72	1.26	98.97	123.44	1.60	98.75
	500	500.36	1.68	100.07	494.88	1.99	98.98
	1000	987.21	1.82	98.72	1001.708	2.02	100.17
	32	31.37	1.47	98.02	32.09	1.99	100.27
Rhodamine B	125	124.42	1.49	99.54	125.57	2.25	100.46
	500	493.54	1.92	98.71	498.80	2.10	99.76
	1000	1003.65	1.33	100.37	999.94	1.99	99.99
Crystal Violet	32	31.50	1.61	98.43	32.14	1.99	100.44
	125	124.97	1.85	99.97	124.62	2.56	99.67
	500	499.75	1.74	99.95	508.51	2.19	101.70
	1000	996.11	1.45	99.61	1001.24	2.76	100.12

**Table 4:** Recovery studies for the determination of methylene blue, rhodamine B and crystal violet.

Methylene Blue			Rhodamine B					Crystal Violet			
Spiked (ng mL^−1^)	Found (ng mL^−1^)	RSD (%)	Recovery (%)	Spiked (ng mL^−1^)	Found	RSD (%)	Recovery (%)	Spiked (ng mL^−1^)	Found (ng mL^−1^)	RSD (%)	Recovery (%)
64	62.79	1.56	98.11	64	62.93	0.66	98.33	64	63.58	2.30	99.34
125	123.25	1.34	98.60	125	123.94	1.89	99.15	125	124.41	2.82	99.53
250	248.09	2.59	99.23	250	248.64	2.15	99.45	250	248.61	1.77	99.44
1000	990.97	2.21	99.09	1000	989.15	1.39	98.91	1000	995.54	0.67	99.55

**Table 5:** Optimization of the flow rate of solid-phase extraction for the determination of methylene blue (MB), rhodamine B (RB) and crystal violet (CV).

Flow Rate	Concentration of Eluent	% of MB Found in Eluate	% of RB Found in Eluate	% of CV Found in Eluate
0.5 mL min^−1^	500 ng mL^−1^	Below LOQ	Below LOQ	Below LOQ
0.75 mL min^−1^	500 ng mL^−1^	Below LOQ	Below LOQ	Below LOQ
1.0 mL min^−1^	500 ng mL^−1^	11.3%	14.6%	12.9%
2.0 mL min^−1^	500 ng mL^−1^	40.1%	54.6%	49.8%
3.0 mL min^−1^	500 ng mL^−1^	70.1%	80.2%	75.9%

**Table 6:** Determination of methylene blue, crystal violet and rhodamine B in environmental samples.

S. No	Type of Sample	Methylene Blue (ng mL^−1^)	Rhodamine B (ng mL^−1^)	Crystal Violet (ng mL^−1^)
01	Laundry sample 1	390	320	270
02	Laundry sample 2	430	410	380
03	Textile dyeing unit waste sample 1	5560	1030	3890
04	Textile dyeing unit waste sample 2	4020	1920	4360
05	Printing press	1930	ND *	2090
06	Domestic supplies	ND *	ND *	ND *
07	Bottled drinking water	ND *	ND *	ND *
08	Irrigation supply water	ND *	ND *	ND *

* ND: not detected
